# Recommendations for extracellular vesicle miRNA biomarker research in the endometrial cancer context

**DOI:** 10.1016/j.tranon.2022.101478

**Published:** 2022-07-09

**Authors:** Emily Paterson, Cherie Blenkiron, Kirsty Danielson, Claire Henry

**Affiliations:** a Department of Obstetrics, Gynaecology and Women's Health, University of Otago, Wellington, New Zealand; b Faculty of Medical and Health Sciences, University of Auckland, Auckland, New Zealand; c Department of Surgery and Anaesthesia, University of Otago, Wellington, New Zealand

**Keywords:** Extracellular vesicles, miRNAs, Biomarkers, Endometrial cancer

## Abstract

Endometrial cancer (EC) is the most common gynaecological malignancy in the developed world, and concerningly incidence is rising, particularly in younger people. Therefore, there is increased interest in novel diagnostic and prognostic biomarkers. Extracellular vesicles (EVs) are membrane-bound particles present in bodily fluids that have the potential to facilitate non-invasive, early diagnosis of EC and could aid with monitoring of recurrence and treatment response. EV cargo provides molecular insight into the tumor, with the lipid bilayer providing stability for RNA species usually prone to degradation. miRNAs have recently become a focus for EV biomarker research due to their ability to regulate cancer related pathways and influence cancer development and progression. This review evaluates the current literature on EV miRNA biomarkers with a focus on EC, and discusses the challenges facing this research. This review finally highlights areas of focus for EV miRNA biomarker research going forward, such as standardization of normalization approaches, sample storage and processing, extensive reporting of methodologies and moving away from single miRNA biomarkers.

## Graphical abstract

**Figure:**
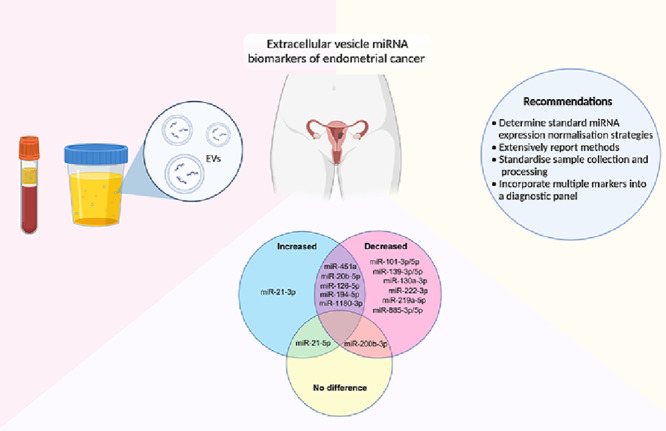

## Introduction

Endometrial cancer (EC) is the most common gynaecological cancer worldwide, and is one of the few cancers with increasing incidence [1,2]. Patients generally present with the symptom of abnormal uterine bleeding resulting in most ECs being detected early, with around 75% of patients being diagnosed with stage I cancer [3].

EC diagnosis is normally through pipelle biopsy, an invasive and painful procedure that is often repeated due to poor tissue collection [4]. The cancer is then classified by stage, grade and histological subtype, and more recently, also by molecular subtype [5]. Current gold-standard treatment for EC, including early stage cancers, involves a total hysterectomy and bilateral salpingo-oophorectomy, resulting in fertility loss and early entry into menopause [6]. While unsuitable for more aggressive histological (serous) and molecular (p53 abnormal) subtypes, progestin-based treatment is gaining traction as alternative treatment for early stage, low grade ECs, however pipelle biopsies are required every three to six months during progestin therapy to monitor treatment response [7]. Biomarkers present in bodily fluids provide an opportunity to reduce the need for these invasive biopsies, lessening the burden on both patients and clinicians. Liquid biopsy may facilitate a simpler method of diagnosis and subtyping on EC suspicion, less invasive monitoring of treatment response and disease progression, as well as potentially predicting response to treatment and recurrence.

**Fig. 1:**
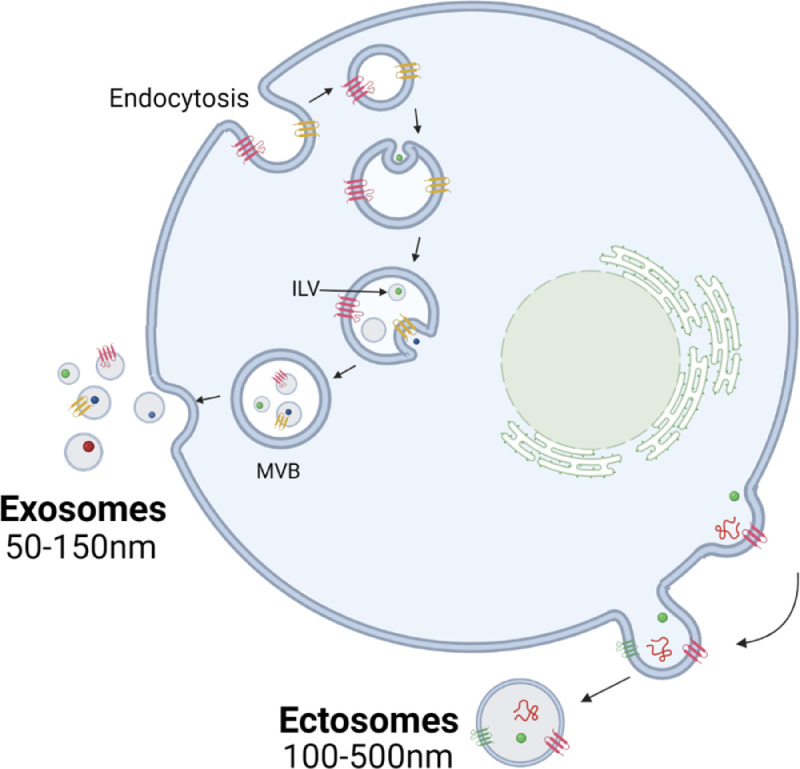
Biogenesis of the two main classes of extracellular vesicles, exosomes and ectosomes. Exosomes are formed through the endocytic pathway, through the invagination of the endosomal membrane to form intraluminal vesicles (ILVs) which are released into the extracellular space following the fusion of the multivesicular body (MVB) and plasma membrane. Ectosomes, also referred to as microvesicles or microparticles, are formed through the outward budding of the plasma membrane. (Created with BioRender.com).

Extracellular vesicles (EVs) are membrane-bound particles that cannot replicate and are actively released from most cell types. EV is a general term used to describe a range of particles which differ by biogenesis [8]; exosomes, formed from the endocytic pathway and ectosomes, derived from the plasma membrane (Fig. 1). As characteristics such as size, density and protein markers show significant overlap [9], most isolation methods enrich a heterogeneous population of EVs, yet many papers still incorrectly use the term ‘exosome’. For the purpose of this review, we will refer to all preparations in previous studies as EVs. EV cargo includes molecules such as DNA, proteins and various RNA species, and the transfer of these molecular messengers is a critical form of cell communication that can alter the phenotype of the recipient cell. Their abundance in a range of bodily fluids [10], [11], [12] along with the long-term stability even after freeze-thaw cycles provided by the lipid bilayer makes EVs attractive as biomarkers for clinical use [13]. Alternative biomarkers such as circulating tumor cells (CTCs) or circulating tumor DNA (ctDNA) can be difficult to detect particularly if the cancer is early stage. With most ECs being diagnosed at stage I [3], EVs have unique value as biomarkers of EC. miRNAs are short, single stranded non-coding RNAs that predominantly act as post-transcriptional regulators of gene expression and are often dysregulated in disease. As the most abundant non-coding RNA species associated with EVs [14] and their ability to modify proliferative and metastatic pathways to contribute to cancer development, miRNAs have become of interest for biomarker research [15]. miRNAs are selectively packaged into EVs for cell communication and show differing abundance profiles in EVs and cell-free plasma [16], suggesting that EV miRNAs may be more tumor specific than free circulating miRNAs and therefore a superior biomarker. As such, EVs have provided a new avenue for miRNA biomarker research to explore.

This review discusses the current literature of EV miRNA biomarkers in EC diagnosis, highlights the current limitations in general EV miRNA research and provides recommendations for research in this overall field going forward.

## EV miRNA biomarkers of endometrial cancer

miRNAs have the potential to act as diagnostic and prognostic biomarkers of EC, as demonstrated in a multitude of studies [15,[17], [18], [19], [20]]. EVs facilitate cellular crosstalk and show selective miRNA packaging, and, as a result, EV, tissue and extracellular miRNA biomarkers can have differing performance and predictive ability [21]. Fan et al. [17] demonstrated this in the EC context, identifying a six miRNA whole serum signature with diagnostic potential, with only one of these miRNAs showing differential abundance in enriched serum EVs. EVs are therefore a novel source of biomarkers, differing from circulating miRNAs and provide a new opportunity for biomarker discovery.

Multiple studies have identified EV miRNAs that are differentially abundant in EVs between EC patients and healthy controls. Table 1 summarizes the main characteristics of EC EV miRNA biomarker studies carried out to date.

**Table 1:** EV miRNA biomarker studies in endometrial cancer.

Refs.	Cohort (n)	FIGO stage	Histology	EV biosource	Isolation method	Test platform	Normalisation strategy	miRNAs screened	Outcomes
Zavesky et al. [22]	Patients *n* = 10 Controls *n* = 19	Stage I: 1 Stage III: 2 Unknown: 7	Type I: 9 Type II: 1	Urine	Urine Exosome RNA Isolation Kit (Norgen Biotek)	RT-qPCR	Geomean of all miRNAs investigated	9	Difference in diagnostic performance of miRNAs in urine supernatant vs EVs. No miRNAs investigated were differentially abundant in EVs in EC vs controls.
Srivastava et al. [23]	Patients *n* = 22 Controls *n* = 5	Not specified	Not specified	Urine	UC: 10,000 g 30 min 100,000 g 2 h	microRNA PCR array & RT-qPCR	Not specified	84	57 miRNAs had detectable expression within EVs. miR-200c-3p most enriched miRNA in EC EVs, expression validated with RT-qPCR.
Roman-Canal et al. [24]	Patients *n* = 25 Controls *n* = 25	Stage I: 13 Stage II: 4 Stage III: 6 Unknown: 2	Endometrioid: 20 Other: 5	Peritoneal lavage	UC: 300 g for 10 min 2500 g for 20 min 10,000 g 30 min 100,000 g for 2 h, twice	microRNA PCR array	Endogenous controls: miR−150−5p, let−7g-5p, miR−598−3p, and miR−361−3p	754	Identified 114 differentially abundant miRNAs between cases and controls. Assessed biological processes and molecular functions the predicted targets of the differentially expressed miRNAs are involved in.
Zheng et al. [25]	Patients *n* = 100 Controls *n* = 100	Stage I & II: 54 Stage III & IV: 46	Not specified	Serum	UC: 3000 g for 10–20 min 1500 g for 25 min	RT-qPCR	Endogenous controls: miR-214-5p & miR-16-5p	2	miR-93 and miR-205 were differentially abundant in EVs between EC and controls. >3 fold miR-93 and <0.07 fold miR-205 abundance were each independently associated with reduced overall median survival.
Zhou et al. [26]	Discovery: Patients *n* = 25 Controls *n* = 31 Validation: Patients *n* = 115 Controls *n* = 87	Discovery Stage I: 17 Stage II: 5 Stage III: 2 Stage IV: 1 Validation Stage I: 82 Stage II: 15 Stage III: 17 Unknown: 1	Discovery Type I: 18 Type II: 4 Unknown: 3 Validation Type I: 51 Type II: 11 Mixed: 48 Unknown: 5	Plasma	ExoQuick Exosome Precipitation Solution (System Biosciences)	Small RNA sequencing & ddPCR	Endogenous controls: let-7b-5p & miR-26a-5p	N/A	49 differentially abundant miRNAs identified. Digital droplet PCR validated upregulation of miR-106b-5p, miR-107, and miR-15a-5p in EC. miR-15a-5p associated with muscular infiltration depth (≥1/2), tumor size (≥ 10 cm^3^), and reproductive hormone levels.
Fan et al. [17]	Patients *n* = 30 Controls *n* = 30	Stage I: 27 Stage II, III, IV: 3	Not specified	Serum	ExoQuick Exosome Precipitation Solution (System Biosciences)	RT-qPCR	Endogenous control: U6	6	Expression patterns of candidate miRNA biomarkers between total serum and EVs was only consistent for miR-20b-5p.

EC – endometrial cancer, EV – extracellular vesicle, FIGO – International Federation of Gynaecology and Obstetrics, RT-qPCR – quantitative reverse transcriptase polymerase chain reaction, UC – ultracentrifugation.

Two studies stand out for evaluating the abundance of a large number of miRNAs within EC EVs. Zhou et al. [26] identified 49 differentially abundant miRNAs using small RNA sequencing, and Roman-Canal et al. [24] identified 114 differentially abundant species using a more targeted 754 miRNA polymerase chain reaction (PCR) microarray. Zhou et al. [26] went on to demonstrate the diagnostic ability of miR-15a-5p, with an area under the curve (AUC) of 0.813 for distinguishing Stage IA cancers from healthy controls. This was increased to 0.869 when miR-106b-5p and miR-107 were also included, highlighting the value of using multi-analyte measurements for miRNA biomarkers. miR-15a-5p was linked to pathological features, with elevated levels associated with increased myometrial invasion depth (≥1/2) and larger tumor volume (≥ 10 cm^3^), suggesting miR-15a-5p may be a marker of more aggressive cancers. As such, miR-15a-5p may have potential as a prognostic marker but has yet to be studied more extensively with patient outcome data.

Zhou et al. utilized serum and precipitation-based EV isolation while Roman-Canal et al. investigated peritoneal lavage EVs isolated using differential ultracentrifugation. Despite these differences, there were eight miRNAs that showed consistent directional change with miR-21-5p increased and miR-101-5p, miR-130a-3p, miR-139, miR-200b-3p, miR-219a-5p, miR-222-3p and miR-885 reduced in EC EVs. Four miRNAs miR-126-5p, miR-194-5p, miR-451a, miR-1180-3p had opposite expression patterns observed, showing increase in one study and decrease in the other. miR-451a levels are significantly altered by the degree of haemolysis of blood samples [27], so this may not be a true marker of EC but instead a result of sample processing variability. Table 2 summarizes the expression patterns of miRNAs with evidence of differential abundance validated in more than one EV EC biomarker study.

**Table 2:** Expression patterns of EV miRNAs in EC compared to healthy controls with differential abundance validated in more than one study.

miRNA	Upregulated	Downregulated	No difference
miR-20b-5p	Fan et al. [17]	Roman-Canal et al. [24]	
miR-21-	-3p Zhou et al. [26] -3p Roman-Canal et al. [24] -5p Roman-Canal et al. [24]		-5p Zavesky et al. [22]
miR-101-		-5p Zhou et al. [26] -3p Roman-Canal et al. [24]	
miR-126-5p	Roman-Canal et al. [24]	Zhou et al. [26]	
miR-130a-3p		Zhou et al. [26] Roman-Canal et al. [24]	
miR-139-		-5p Roman-Canal et al. [24] -3p Zhou et al. [26]	
miR-194-5p	Zhou et al. [26]	Roman-Canal et al. [24]	
miR-200b-3p		Roman-Canal et al. [24] Zhou et al. [26]	Zavesky et al. [22]
miR-219a-5p		Roman-Canal et al. [24] Zhou et al. [26]	
miR-222-3p		Roman-Canal et al. [24] Zhou et al. [26]	
miR-451a	Zhou et al. [26]	Roman-Canal et al. [24]	
miR-885-		-5p Roman-Canal et al. [24] -3p Zhou et al. [26]	
miR-1180-3p	Zhou et al. [26]	Roman-Canal et al. [24]	

Fan et al. [17] – serum EVs.

Roman-Canal et al. [24] – Peritoneal lavage EVs.

Zavesky et al. [22] – Urine EVs.

Zhou et al. [26] – Plasma EVs.

Of the differently abundant miRNAs present in both of these studies discussed prior, levels of miR-21-5p and miR-200b-3p had been previously investigated in urine EVs with no difference found between EC and controls [22]. However, the small EC cohort of *n* = 10 in their pilot study is a strong limitation. Further contradictory results include Zheng et al. [25] finding miR-93 and miR-205 to be differentially abundant, which has not been replicated in other studies [24,26]. miR-93 was increased and miR-205 was decreased in EVs of stage III and IV patients compared to stage I and II. However, this study does not specify the arm (5p or 3p) of the miRNAs investigated. The dominant arm can differ between tissue types, and each arm can regulate different signaling pathways [28] and thus reporting the miRNA arm investigated is important for accuracy, reproducibility and preventing confusion within the literature. All other studies included in Table 1 describe this information, which is invaluable for drawing comparisons between studies.

Using a PCR microarray, Srivastava et al. [23] found miR-200c-3p was the most enriched miRNA in EC EVs, again isolated from urine. miR-200c-3p has not been found to be differentially abundant in any other EV miRNA studies thus far, but has been found to be upregulated in EC tissue compared to healthy endometrial tissue [29,30]. The authors report large variation in miRNA abundance between donor samples, which may explain why the results are presented as the top 10 ‘most enriched’ miRNAs with no *p*-value presented for the fold change data. Resultingly, the lack of statistical comparisons between expression in cases and controls and the unavailability of the data limits the utility of the study. Of the other miRNAs most abundant in EC EVs in the Srivastava et al. study, increased abundance was also found in one other study for miR-21-5p, while miR-100-5p, miR-26a-5p, miR-26b-5p and miR-125a were found to be reduced in other studies [24,26]. There is no evidence of differential abundance of the remaining miRNAs miR-23b-3p, miR-27b-3p, miR-30c-5p and let-7a-5p in the literature.

The absence of standardized methodologies and heterogeneity in study design (also highlighted in Table 1) contributes to the inconsistent expression patterns reported in the literature, resulting in difficulty in drawing comparisons. The current literature has provided a basis for future studies to begin investigations from, highlighting miRNAs of interest to further research on EC EVs. By evaluating these studies together, their limitations can be used to provide guidance on the best, more standardized approaches for future EC EV miRNA studies.

## Challenges facing endometrial cancer EV miRNA biomarker research

The previously discussed proposed EV miRNA biomarkers of EC need to be interpreted with caution, due to absence of critical methodology details and inconsistent experimental design in some studies. The data previously presented must be considered in the context of these issues and care must be taken when extrapolating results. Specific factors affecting interpretation of current evidence of EV miRNA biomarkers collectively, of relevance to both EC and to general disease biomarker studies, are discussed subsequently.

### EV isolation

There are a wide range of techniques that can be used for isolating EVs from biofluids, which have been reviewed extensively [31], [32], [33]. For clinical use, EV isolation needs to be rapid, easily scalable and cost effective, and the ideal isolation methodology is an area of ongoing debate surrounding EV-based biomarkers. Commercial kits meet these requirements but are plagued by high levels of protein, vesicle-free miRNA and other non-vesicular contamination [34,35]. This creates issues for accurate biomarker measurement and true assignment of the identified miRNAs to the EV-fraction of the biofluid. While ultracentrifugation (UC) is still the most widely used isolation method [36], UC is time consuming and requires expensive specialist equipment, limiting clinical utility. Size exclusion chromatography (SEC) isolates EVs with lower levels of non-specific miRNA contamination [37], but currently has limited scalability. As some isolation methodologies favour both EVs and co-isolated non-vesicular material of differing size and densities [38], the chosen isolation technique impacts on downstream RNA profiling and analysis [39], [40], [41]. Even minor changes to methods due to differing interpretation of the same protocol can impact on the subfractions of EVs and co-contaminants isolated [42]. Therefore, a clinical test will require standardization of the entire assay as the biomarkers may be specific to the fraction isolated by the exact methodology used. Current methods have advantages and drawbacks which must be balanced, and a decision made about which factors are priorities, whether that be purity, yield, cost or efficiency. Technological advancement could reduce the number of steps involved in EV isolation to minimize the points at which variation can be introduced, which is appealing for clinical use. This includes a move towards automation of EV isolation, such as automated fraction collectors for isolation using SEC. However, automation is not possible for some widely popular isolation methods including UC. Other recent innovations such as chip biosensors (reviewed by Wang et al. [43]) have the potential to improve EV isolation and miRNA detection as a clinically accessible assay.

### Normalization strategies

All EC EV miRNA studies (Table 1) utilized quantitative PCR (qPCR) assays, digital or real-time, to measure EV miRNAs, either as a discovery or validation methodology. Most studies used endogenous reference gene sequences to normalize miRNA abundance and remove differences due to sample input or experimental factors. The most critical feature of an endogenous reference sequence is stable presence across the conditions investigated to achieve accurate abundance profiles. This includes stable miRNA levels between cases and controls, unaffected by cancer grade and stage. Selecting appropriate references for miRNA studies continues to be challenging, with no consensus on the most appropriate miRNA controls in EC [44]. Selective miRNA packaging into EVs means miRNA reference sequences used for cell lines or tumor tissue cannot be assumed to be suitable for use in EV studies, and require within sample validation prior to application [45].

To date, no studies of EC EV miRNA reference sequences have been conducted. Analyzing data across studies can be a useful way of identifying consistently expressed species. However, as there is currently only one EC EV study [26] with data submitted to a public repository, this approach is not possible. It is important to note that as the stability of miRNAs would be impacted by EV isolation method, so studies must be similar when making comparisons. U6 and miR-16-5p are commonly used EV miRNA reference sequences, also used in EC EV studies [17,25]. U6 is a small nuclear RNA that forms part of the spliceosome and may be an inherently unsuitable reference due to: longer length compared to mature miRNAs which may influence packaging into EVs [46], differing stability compared to miRNAs [47], debate around U6 presence within EVs due to cellular localization to the nucleus [48], and evidence of differential abundance within various cancer EVs [49,50]. miR-16-5p has reportedly stable expression in various disease state EVs [51], [52], [53], but stable abundance, where abundance accounts for both cellular expression and subsequent packaging into released EVs, in EC EVs has yet to be demonstrated. As further reference candidates, small RNA sequencing identified miR-26a-5p and let-7b-5p as having stable levels in plasma EVs from EC cases and controls [26]. With validation to confirm stable levels in different experimental conditions these alternative miRNAs may be useful references for the EC EV field. However, each study may need to define their own reference sequence or utilize alternative normalization strategies.

Another approach is to normalize to the mean abundance of all miRNAs investigated, which is useful when assaying large numbers of miRNAs during pre-clinical research. However, this is not appropriate when fewer than fifty miRNAs are investigated [54], so is not a viable option in a clinical setting or during the validation phase of miRNA studies. MiRNA abundance can also be normalised to EV particle number, or EV protein yield, however with current methods of isolation and EV characterization, these numbers are often contaminated by non-vesicular material so do not represent 'pure’ EVs [55].

Alternative quantification strategies may be more appropriate for EV miRNA studies. EVs have a small capacity for carrying miRNA molecules, and the number of informative EVs is often elevated in cancer patients compared to controls. As such, the number of miRNAs per EV may not be the most appropriate measure and normalization strategies such as endogenous controls or RNA quantity input may reduce the diagnostic power. An alternative strategy is to therefore use exogenous spike-ins of non-human sequences, which provides normalization of technical factors but does not mis-account for pathological differences in starting miRNA concentration.

The lack of standardised approaches for EV miRNA abundance analysis affects reproducibility. Quality normalization strategies will be required for clinical use of EV miRNA biomarkers and determining these while research is still in pre-clinical phases will aid reproducibility and translation into clinical cohorts. Selection of an appropriate normalization strategy is critical, as the approach taken can change the results found [56]. The most appropriate approach may however differ between studies with varying study designs, and therefore the chosen strategy should be selected carefully.

### Study design heterogeneity

The EC EV miRNA studies carried out thus far are extremely varied in design, leading to the inconsistent results, highlighted in Table 2, and creating challenges with drawing comparisons between studies.

A key area of difference between the studies is the biofluid used to isolate EVs from. While EVs have been isolated from almost all bodily fluids, the main sources commonly used include urine and blood (both serum and plasma) [36]. Urine is very accessible, with collection simple, pain-free and non-invasive. However, urine composition shows high variation, is easily influenced by dietary, medicinal and diurnal changes and is particularly susceptible to collection error by patients [57]. Meanwhile, serum and plasma can differ significantly, and both are commonly contaminated with lipoproteins which are affected by fasting state. The relationship between BMI and increasing lipoprotein concentration [58] indicates this contamination may be a particular challenge for EC EV biomarkers, with high rates of obesity in EC patient populations; importantly this is the population in whom increasing incidence is observed [59]. Other co-morbidities associated with EC including type II diabetes will also change the miRNAs and EVs present within the blood [60]. EV miRNAs have been isolated from peritoneal lavage in one EC study [24], and the proximity to the uterus may result in enriched levels of tumor EVs in peritoneal fluid, particularly if metastatic disease is present. The majority of EVs within serum and plasma are derived from blood cells [61], reducing the ability to detect miRNAs within relatively rare tumor EVs, which could be partly overcome by the use of peritoneal lavage. Increased tumor burden associates with increased secretion of tumor EVs [62], but with the majority of ECs being Stage I the proportion of tumor EVs within biofluids such as blood is likely to be low. However, non-tumor EVs can still provide valuable insight into the immune and inflammatory responses occurring systemically and be a good non-tumor derived biomarker of disease. As peritoneal lavage is obtained during surgery, the difficulty of obtaining this biofluid renders it unsuitable for diagnostic biomarkers. Instead, searching for biomarkers in this potentially enriched biofluid should be then followed by investigating candidates in more readily available sources such as blood. Other sources that may be enriched with tumor derived EVs include uterine blood, particularly relevant due to the high incidence of abnormal uterine bleeding in EC patients [63], and uterine lavage, but these biofluids have not yet been investigated for EV miRNA cargo. With no current consensus on the optimal biofluid for EV EC biomarkers, many sources remain valid choices.

Another important source of study heterogeneity is the EC cohort, particularly the stage of the cancers. EC cohorts in studies by Zhou et al. [26] and Fan et al. [17] were made up of mostly Stage I patients, which reflects the over representation of Stage I cancers seen in clinical EC patient populations, and is ideal for investigating biomarkers for early diagnosis. Meanwhile, the cohort in the study by Zheng et al. [25] was comprised of almost half stage III and IV cancers. Some report the number of patients with each stage of cancer separately [22,24,26], while other studies clustered stages together [17,25], or did not report stage at all [23]. As cohort sizes in these studies are limited, there are only often a handful of patients with Stage II or above cancers making this clustering logical. However, this limits the ability to assess the impact of stage on miRNA abundance. Other highly relevant clinicopathological information that is poorly reported is cancer subtype, with only one study detailing histological subtype [24], two using the old Bokhman classification system [22,26] and no studies reporting molecular subtype. Without this important information, associations with histological or molecular subtypes cannot be investigated. Furthermore, medical comorbidities such as diabetes and hypertension are also underreported, even though they are highly prevalent in the EC patient population [64]. The lack of patient outcome data in most of these studies prevents the investigation of prognostic biomarkers. Small cohorts with inherent heterogeneity are common due to patient sample availability and financial constraints, resulting in biological noise and a reduced power to identify robust and sensitive biomarkers with true differences. All clinical variables should therefore be reported, and their possible impact should be considered and used to adjust study design accordingly.

## Future directions

Variations in study design, normalization strategies and EV isolation methodologies have contributed to the conflicting results found between EC EV miRNA biomarker studies. These factors, along with other challenges associated with EV-based biomarkers, have hindered the validation and clinical implementation of candidate EV miRNA biomarkers for all disease states, including EC. As such, we subsequently highlight areas that require attention to improve reproducibility of future research and subsequent advancement into clinical practice.

(1) Determine standard normalization approaches

A factor for consideration is translating pre-clinical findings into clinically implementable tests. PCR-based assays remain the test with the greatest clinical applicability, with RNA sequencing and microarrays both costly and time-consuming. For the clinical implementation of a PCR test for EV miRNA biomarkers, the absence of standard endogenous reference sequences for normalization is an issue, as previously discussed. The impact of isolation methodology and EV source on the population of EVs isolated means stable abundance of candidate reference sequences needs to be validated for each isolation methodology and biofluid used. Thus, alternative approaches discussed earlier such as exogenous spike-ins may be more suitable for EV biomarkers. Normalization strategies for any assay, PCR-based or otherwise, should also be standardized as the approach taken can affect the results and thus impact on reproducibility.

(2) Extensive reporting of methodology

The Minimal Information for Studies of Extracellular Vesicles (MISEV) guidelines [8,65] have greatly aided the EV field, outlining the key requirements for EV work and encouraging extensive reporting of methodology to increase reproducibility. The methods selected should also be appropriate and should rigorously evaluate EV isolations prior to downstream use to fulfill EV characterization obligations. Current results attributed to EVs, or in some studies to the exosome subset, are likely to be context dependent and specific to the parameters and experimental design used in the study, and therefore interpretations may be erroneous due to inappropriate design. Thus, sample collection, processing and methodologies must be extensively reported to understand the context in which the results are set, to allow others working in the field to evaluate the authenticity of the result and to enable future validation.

Further recommendations of a similar nature to the MISEV guidelines are needed, providing processing and reporting requirements for the various biofluids commonly used for EV isolation, and for downstream analyses such as miRNA analysis. This would benefit scientists new to EVs, while also aiding the translatability of all EV research. The International Society of Extracellular Vesicles (ISEV) have begun this work with papers providing recommendations for working with urine [66] and blood EVs [67], and taskforces are working on similar guidelines for other contexts [68]. Other groups such as Extracellular RNA Communication Consortium are also completing valuable work in this space [69]. In the absence of standardization, extensively reported methodologies with thorough evaluation of isolated EVs is essential to improve reproducibility and translatability of EV biomarker studies.

(3) Standardize sample processing and storage

There are an immense number of pre-analytical factors that are recommended to be reported on, as outlined in a position paper published following the 2012 ISEV workshop [70]. Almost a decade on, many EV studies fail to report critical sample collection, processing and storage details and standardization has yet to be implemented in EV research.

The extent of standardization must be kept reasonable without excessive collection and processing specifications, so the implementation of these requirements is feasible. However, a certain level of standardization will be required to ensure the reliability of the EV biomarker. Many pre-analytical factors which affect EV yield can be easily standardized such as storage temperature, anticoagulants, measurement of haemolysis and centrifugation speeds [13,71,72]. More challenging factors include venepuncture technique [73], sample agitation [74], fasting state [75] and time between collection and processing [13]. Standardization of these factors across samples within a study is essential, so the comparisons made are valid. These issues are not unique to EV-based biomarkers, with the National Biomarker Development Alliance (NBDA) citing insufficient control of pre-analytical parameters as the one of the leading hindrances in biomarker discovery [76]. Prioritizing the reasonable standardization of sample processing and storage factors sooner rather than later will aid the translation of pre-clinical studies. Standardization will also facilitate multi-center studies and enable collaboration to create larger cohort sizes with increased power to detect true biomarkers.

(4) Move away from single miRNAs as diagnostic biomarkers

A single EV miRNA is unlikely to have utility as a sole diagnostic biomarker of EC. A single EV miRNA is often dysregulated in several cancer types, for example miR-21-5p is dysregulated in liver [77], bladder [78] and breast cancer [79]. While potentially useful as a suspicion marker for cancer screening, this miRNA lacks the specificity required for EC-specific diagnosis. There is also often contradictory evidence within the same disease state, with the proposed prognostic EC biomarker miR-205 being associated with both decreased and increased overall survival [18,30]. Further complicating things, the regulation of target genes can differ within the same tissue type depending on other molecular factors [80]. Therefore, combining various molecular information should improve specificity for EC diagnostics, shown by Zhou et al. with greater predictive ability when combining three EV miRNAs compared to a single miRNA [26]. Ensuring specificity to EC is important so that appropriate treatment is provided in a timely manner, and to avoid misdiagnosis and subsequent unnecessary testing and treatments. This may involve integrating other biomarkers, such as cell-free miRNAs, DNA or proteins, into a panel with EV miRNAs. Integrating EV miRNAs with traditional blood-based protein markers has already been demonstrated to improve sensitivity and specificity in EC [26] and other cancers [81,82]. Other EV studies have identified EV proteins that have potential as biomarkers, such as elevated EV LGALS3BP being associated with EC [83,84]. Thus far, miRNAs are the most widely researched EV non-coding RNA species investigated, with only one study investigating EV circular RNA (circRNA) biomarkers of EC [85]. Further research into other regulatory RNA species as biomarkers is warranted, as functional studies have identified EV circRNAs and long non-coding RNAs (lncRNAs) that may contribute to EC progression [86] and radiation treatment resistance [87]. Combinations of non-coding RNA species may provide unique insight into regulatory networks that contribute to EC pathogenesis, which could form the basis of a biomarker panel. Well-designed future studies could also identify EV biomarkers that distinguish the histological and molecular subtypes of EC, enabling risk stratification of EC cases. As such, future research should focus on investigating combinations of molecules with predictive ability specific to EC.

## Conclusions

EVs provide an exciting opportunity to progress miRNA biomarker research, with their stability and abundance making EVs ideal for use clinically as diagnostic biomarkers. Currently, there are only a handful of studies focusing on EV-based miRNA biomarkers in EC. Results thus far have been inconsistent, with limited reproducibility and translatability due to disparate study design and incomplete methodological reporting. Well designed and large-scale future studies with robust methodologies are required to identify and validate candidate biomarkers. An important focus for the EV field is improving reproducibility through thorough methodological reporting and standardized approaches for expression normalization and sample processing. With further research into the areas outlined, EV miRNA biomarkers have great potential to facilitate simpler, accurate and more accessible EC clinical diagnostics.

## Declaration of Competing Interest

The authors declare that they have no known competing financial interests or personal relationships that could have appeared to influence the work reported in this paper.
